# Report of Committee on Revision of Constitution and By-Laws

**Published:** 1894-11

**Authors:** W. B. E. Miller, Leonard Pearson

**Affiliations:** Philadelphia, Pa.; Philadelphia, Pa.


### REPORT OF COMMITTEE ON REVISION OF CONSTITUTION AND BY-LAWS.

THIRTY-FIRST ANNUAL MEETING, UNITED STATES VETERINARY MEDICAL ASSOCIATION.

PHILADELPHIA, PA., Sept., 1894.

*To the President and members of the United States Veterinary Medical Association:*

GENTLEMEN:—Your Committee on Revision of the Constitution and By-Laws would most respectfully report that, because of the failure to secure articles of incorporation from Congress, we are unable to make a full and complete report, such as will be necessary when the Association becomes an incorporated body. A few important changes are, to the minds of your Committee, very essential, and we would recommend that they receive favorable consideration at this time. To wit, to change Article III, second line, of the Constitution, by striking out the word "Recording," before Secretary. Also to add to the word "three" (3) before Vice-President, and to add the letter s to the word President, making it read thus: "Three Vice-Presidents, one from the East, one from the Central, and one from the Western section of the country."

To change Article I, Chapter 2d, of the By-Laws, third line, by inserting the words "and dues" after the word "fees"; making it read thus: "All applications and fees and dues for membership."

To change Article IV, same chapter, by striking out the word "Recording."

To change Article V, same chapter, so as to make it read as follows: "He shall by virtue of his office be a member of the Publication Committee, and shall receive an annual salary of two hundred dollars."

To change Article II, Chapter 3, by adding to it the following words: "Or as may be ordered by the President, attested by the Secretary."

To change Article II, Chapter 4, by erasing the words "in six months," and adding or substituting after the word "once" the following: "A year upon the call of the President at any time prior to the annual meeting." To add to this chapter, Article IX, as follows: "The President shall have the power to order the payment of any or all bills that may be presented to the Secretary, during the year prior to the annual meeting, if in his judgment they are true and correct, and, if he deems it wise, to make immediate payment of the same. All such expenditures, however, must be accounted for at the annual meeting of the Finance Committee."

To change Article VII, Chapter 5, after the word "Committees," by adding the words, "Except the Committee on Publication, of which the Secretary shall be an active member."

To change Article I, Chapter 6, adding after the word "voucher" the following: "and accompanied by the initiation fee."

To change Article V, by striking out all after the words, "within one year," up to the word "sign," and add to the section the words, "If he fails to do so within this time, he ceases to be a member."

To change Article I, Chapter 7, after the word "proposed," in the fourth line, insert the words "which applicant shall be presented to the Comitia Minora, and acted upon at the next meeting."

To change Article I, Chapter 8, by the word five.

To change Article II, same chapter, by erasing the word

"two (2)," and substituting the word "five (5)," payable in advance."

To change Article I, Chapter 10, by adding the letter "s" to the word "day," in the second line.

To change Article II, Chapter 10, by adding the letter "s" to the word "Vice-President."

To change Article VI, same chapter, by adding the letter "s" to the word "Vice-President," and by erasing the word "Censor," and substituting the words "Past President," and by adding to the section the following words: "In case none should be present, the Association shall elect a President pro tempore, from the floor."

Respectfully submitted,

W. B. E. MILLER,

LEONARD PEARSON.

